# Metal–Organic Framework (MOF)-Derived Materials for Triethylamine Gas Sensing Application for Environmental Monitoring: Recent Advances and Future Perspectives

**DOI:** 10.3390/s26144587

**Published:** 2026-07-20

**Authors:** Khursheed Ahmad, Chellakannu Rajkumar, Tae Hwan Oh

**Affiliations:** School of Chemical Engineering, Yeungnam University, 280 Daehak-Ro, Gyeongsan 38541, Republic of Korea

**Keywords:** MOF-derived materials, triethylamine, gas sensor, environmental monitoring

## Abstract

Metal–organic framework (MOF)-derived materials have recently emerged as promising sensing materials because of their tunable composition, porous architecture, high surface area, and defect-rich structures. Therefore, MOF-derived materials have significantly attracted the scientific community to design and fabricate triethylamine (TEA) gas sensors. TEA is a toxic, volatile, and malodorous amine that is widely released from industrial processes, food spoilage, and environmental sources. The selective and sensitive detection of TEA is of great importance for health, safety, and environmental monitoring. Previous years have witnessed rapid growth in the development of MOF-derived materials based on TEA gas sensors. This review critically evaluates recent progress in the fabrication of MOF-derived metal oxides, mixed-metal oxides, doped systems, noble-metal-functionalized materials, carbon-containing composites, MXene-integrated architectures, and heterojunction-based TEA gas sensors. The response, selectivity, stability, and sensing mechanisms for TEA gas sensors are discussed. Furthermore, challenges and perspectives are discussed. We believe that this review may be beneficial for those actively working in the fabrication of MOF-based TEA gas sensors.

## 1. Introduction

Triethylamine (TEA) is a volatile organic amine that is widely used in chemical manufacturing, pharmaceuticals, pesticides, dyes, resins, corrosion inhibitors, and organic synthesis [1,2,3,4]. Although TEA is an important industrial reagent, it is toxic, flammable, and has an unpleasant fish-like odor [5,6,7]. Exposure to TEA can cause irritation of the eyes, skin, and respiratory tract, while prolonged or high-level exposure may lead to more serious health effects [8,9,10]. In addition to safety concerns, TEA is also associated with food spoilage, especially in fish and seafood products, because volatile amines are produced during the degradation of proteins and nitrogen-containing compounds [11]. Therefore, the reliable detection of TEA is important not only for industrial leakage monitoring but also for environmental protection, indoor air quality control, and food freshness evaluation [12].

Several analytical methods such as gas chromatography [13,14], mass spectrometry [15], and ion mobility spectrometry [16,17] have been used for the detection of volatile amines. These methods can provide high accuracy and sensitivity. However, conventional methods usually require expensive instruments, trained operators, complicated sample preparation, and laboratory-based analysis [18]. Such limitations may restrict their use for rapid, on-site, and continuous monitoring. In this context, chemiresistive gas sensors based on semiconducting materials have attracted considerable attention because of their simple device structure, low cost, miniaturization potential, fast response, and compatibility with portable monitoring systems [19,20,21]. In chemiresistive gas sensors, the interactions between TEA molecules and adsorbed oxygen species on the sensing surface change the resistance of the material, allowing the TEA concentration to be detected through an electrical signal.

Metal oxide semiconductors such as zinc oxide (ZnO) [22,23,24], tin oxide (SnO_2_) [25], indium oxide (In_2_O_3_) [26,27,28], iron oxide (Fe_2_O_3_) [29,30], cobalt oxide (Co_3_O_4_) [31], copper oxide (CuO) [32,33], chromium oxide [34,35], nickel oxide (NiO) [36], molybdenum oxide (MoO_3_) [37,38], and tungsten oxide (WO_3_) [39] as well as non-oxide layered semiconductors such as tungsten sulfide (WS_2_) [40] and molybdenum sulfide (MoS_2_) [41]-based materials have been widely investigated for the detection and oxidation of TEA. It is understood that the sensing behavior of the materials can be largely influenced by surface area, porosity, oxygen vacancies (V_O_), crystallinity, particle size, exposed crystal facets, and interfacial charge transfer [42]. Conventional metal oxide-based gas sensors remain limited by poor selectivity, strong humidity dependence, insufficient long-term stability, and sluggish recovery kinetics [43]. The presence of such shortcomings is particularly critical under realistic operating conditions, where water vapor and competing volatile organic compounds can alter surface reactions and compromise sensing accuracy [44]. Accordingly, the development of advanced sensing materials with abundant accessible active sites, rapid gas diffusion channels, strong and preferential TEA adsorption, efficient oxygen activation, and accelerated charge transport is essential for achieving reliable, low-temperature, and selective TEA detection.

Metal–organic frameworks (MOFs) are widely used as precursors for gas-sensing materials because of their porous structures, high surface area, tunable metal centers, and flexible compositions [45]. However, pristine MOFs often show low electrical conductivity and limited thermal stability, which may restrict their direct use in chemiresistive gas sensors. These limitations can be reduced by converting MOFs into derivative materials [46]. MOF-derived synthesis provides a versatile route to fabricate the hollow, hierarchical, mesoporous, and defect-rich gas sensing materials with controllable composition and interfaces. Calcination, pyrolysis, ion exchange, doping, and hybridization can form porous metal oxides, bimetallic or multi-metallic oxides, carbon-containing composites, and heterostructures [47]. The MOF-derived materials generally provide better gas diffusion, more exposed reaction sites, and improved charge transport [48]. In addition, defect engineering, especially V_O_ regulation, improves oxygen adsorption and activation, which are essential for chemiresistive sensing reactions [49]. Doping with foreign metal ions can tune the electronic structure, modify carrier concentration, and enhance the interaction between TEA and the sensing surface [50]. The construction of p–n, n–n, and p–p heterojunctions may also strengthen interfacial charge modulation and enlarge the resistance change during gas exposure [51]. Finally, noble metal decoration and hybridization with conductive components such as MXenes or carbon materials can improve catalytic activity, electron transfer, and low-temperature sensing performance. Previous reviews have addressed MOF-based gas sensors from broad perspectives, including sensing principles, fabrication methods, transduction platforms, conductive MOFs, and toxic-gas detection [51,52,53,54,55,56,57,58,59,60]. However, a focused analysis of MOF-derived materials for TEA sensing is still lacking. These advantages, together with the limitations of conventional gas sensing materials, motivate a focused review of MOF-derived materials for selective and low-temperature TEA detection.

**Scheme 1:**
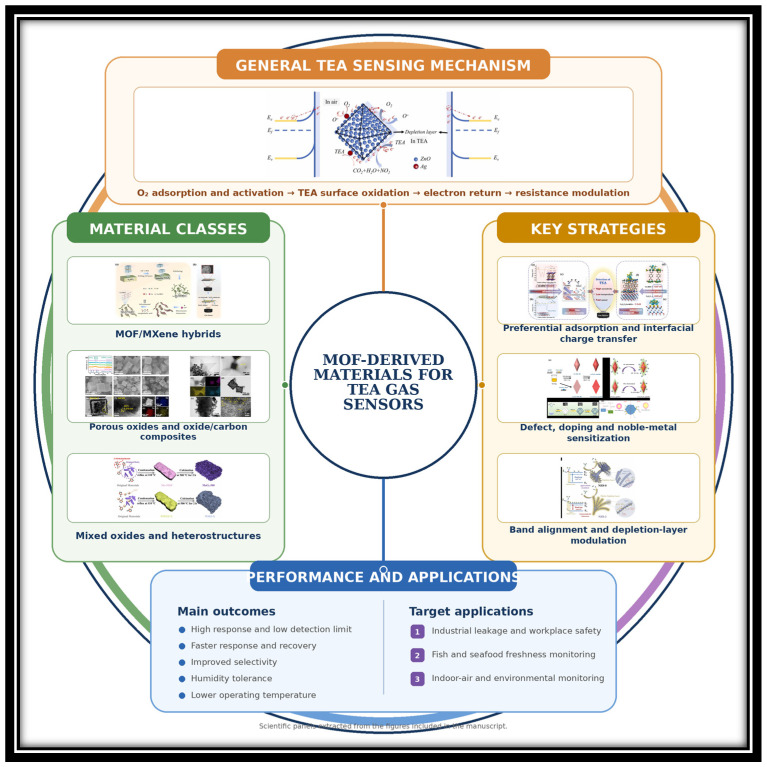
Schematic diagram shows table of content.

This review article summarized recent advances in MOF-derived materials for TEA gas sensing with particular emphasis on material design, structural engineering, sensing performance, and structure–activity relationships. The discussion covers MOF-derived metal oxides, doped oxides, mixed-metal oxides, heterojunction composites, noble metal-modified systems, MXene-assisted materials, and hollow or porous architectures. Finally, current challenges and future perspectives are discussed to guide the development of more practical, low-power, selective, and stable MOF = derived TEA sensors for environmental monitoring, industrial safety, and food quality assessment. Scheme 1 shows the table of contents of this review article.

## 2. Triethylamine Gas Sensors

### 2.1. MOF/MXene-Based Materials

**Figure 1:**
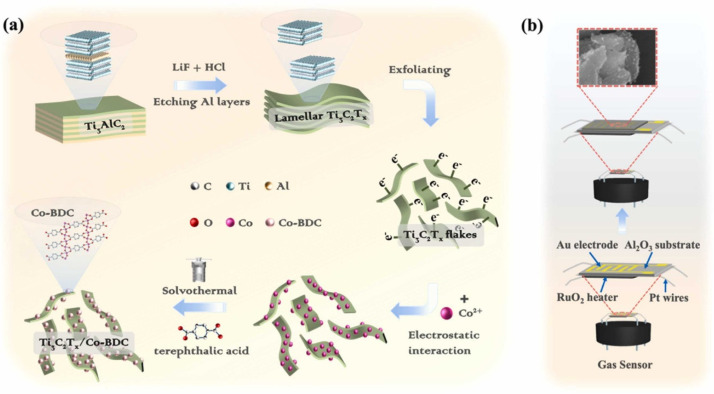
(**a**) Schematic diagram showing the preparation of Ti_3_C_2_T_x_/Co-BDC and (**b**) fabrication of a TEA gas sensor. Reproduced with permission [62].

The sensing performance of MOF-derived materials may be influenced by the interplay between their structural architecture, defect chemistry, and composition. Porous and hollow structures facilitate rapid TEA diffusion and expose a high density of accessible adsorption sites. Defect engineering, particularly the controlled introduction of V_O_, promotes oxygen adsorption and activation, increases the concentration of reactive chemisorbed oxygen species, and modifies the local electronic structure and carrier density. These effects strengthen gas–surface interactions and facilitate charge transfer during TEA oxidation. Further enhancement can be achieved through compositional modulation, including heteroatom doping, heterojunction formation, and noble-metal functionalization, which regulate adsorption energy, catalytic activity, and interfacial charge transport. The resulting synergy accelerates surface redox reactions and amplifies the resistance change. Nevertheless, defect density and adsorption strength must be carefully optimized, as excessive vacancies or overly strong binding may impede desorption, delay recovery, and compromise long-term stability. In previous years, numerous TEA gas sensors were developed using advanced electrode materials. However, MOF-based materials offer several advantages, including high surface area and porosity. Many reports demonstrated the role of MOF-derived materials as a sensing layer for the determination of TEA. In this connection, Liu et al. [61] reported an in situ MOF/MXene-derived strategy to fabricate the chromium oxide–titanium dioxide (Cr_2_O_3_/TiO_2_-X, MCT-X) composite for the monitoring of TEA. In this design, chromium (Cr)-based Materials of Institute Lavoisier-101 (MIL-101) MOF was served as the Cr_2_O_3_ precursor, whereas Ti_3_C_2_T_x_ MXene was used as the sacrificial template to form the TiO_2_. The obtained porous architecture, enlarged surface area, smaller crystallite size, and abundant oxygen vacancies provide more accessible active sites for TEA adsorption and reaction. Among the prepared sensing materials, MCT-2 showed the best performance, delivering a very high response of 450.01 towards 100 ppm TEA at a relatively low operating temperature of 134 °C, along with good selectivity, linearity, repeatability, and stability of 30 days. The sensor could also detect 1 ppm TEA with a response of 9.42, indicating practical potential for early leakage monitoring. The authors also stated that the improved sensing behavior may arise from TEA adsorption on Cr_2_O_3_-rich surfaces and an electron backflow effect at specific Cr_2_O_3_/TiO_2_ crystal-plane contacts as per the density functional theory (DFT) calculations. Huan Liu et al. [62] developed a chemiresistive TEA sensor based on a Ti_3_C_2_T_x_ MXene/cobalt-benzene-1, 4-dicarboxylate MOF (Ti_3_C_2_T_x_/Co-BDC MOF) composite. The Co-BDC MOF was incorporated with layered Ti_3_C_2_T_x_ MXene through in situ interfacial binding strategy, where the conductive MXene sheets improved charge transport while the porous Co-BDC framework supplied abundant adsorption sites for TEA molecules. The synthesis process for the preparation of Ti_3_C_2_T_x_/Co-BDC is illustrated in Figure 1a and the fabrication of a TEA gas sensor using Ti_3_C_2_T_x_/Co-BDC is illustrated in Figure 1b.

**Figure 2:**
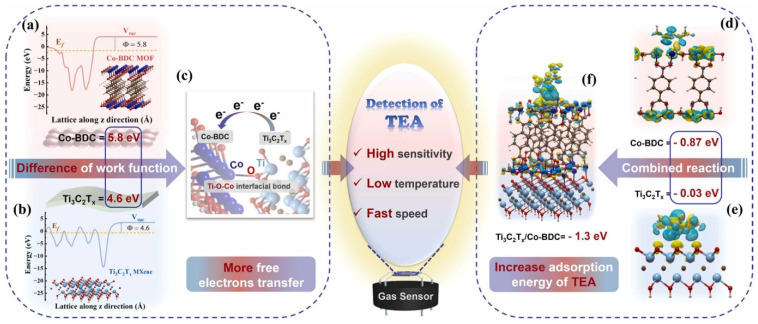
Schematic diagram illustrating the mechanism for improved TEA detection using a Ti_3_C_2_T_x_/Co-BDC MOF-based gas sensor. Work functions of (**a**) Co-BDC MOF and (**b**) Ti_3_C_2_T_x_/Co-BDC MOF. (**c**) Molecular schematic representation of Ti-O–Co bonding/electron transfer process between Co-BDC MOF and Ti_3_C_2_T_x_. Charge density difference (CDD) of TEA adsorbed on (**d**) Co-BDC, (**e**) Ti_3_C_2_T_x_, and (**f**) Ti_3_C_2_T_x_/Co-BDC MOF. Isosurface for Co-BDC = 0.001 e/Å^3^, Ti_3_C_2_T_x_ = 0.001 e/Å^3^, and Ti_3_C_2_T_x_/Co-BDC MOF = 0.0002 e/Å^3^. Reproduced with permission [62].

The optimized Ti_3_C_2_T_x_/Co-BDC composite denoted as CT-2 showed decent sensing behavior and delivered a response of 58.95 toward 50 ppm TEA at a temperature of 110 °C. The sensor also displayed fast response and recovery, good repeatability, long-term stability, and reliable performance under different humidity conditions. In addition, CT-2 has the potential to detect TEA down to 500 ppb, which shows its potential for practical applications. The sensing enhancement may be attributed to the synergistic interaction between Co-BDC and Ti_3_C_2_Tx, which increased the number of active sites, promoted electron transfer, and strengthened TEA adsorption. Additionally, DFT calculations further confirmed that the Ti_3_C_2_T_x_/Co-BDC interface had stronger TEA adsorption than the individual Ti_3_C_2_T_x_ or Co-BDC MOF. Figure 2 explains the DFT-supported sensing mechanism of the Ti_3_C_2_T_x_/Co-BDC-based gas sensor towards TEA. As shown in Figure 2a,b, Co-BDC and Ti_3_C_2_T_x_ exhibit different work functions of 5.8 and 4.6 eV, respectively, which favors electron transfer from Ti_3_C_2_T_x_ to Co-BDC after interfacial contact. Figure 2c further illustrates that the Ti–O–Co interfacial bond acts as an electronic bridge between the two components, forming an internal electric field and accelerating carrier migration. This interfacial electron redistribution is beneficial for faster response and recovery during TEA sensing. The adsorption-energy calculations shown in Figure 2d–f indicate that the Ti_3_C_2_T_x_/Co-BDC composite possesses stronger interaction with TEA than the individual Co-BDC or Ti_3_C_2_T_x_, with more negative adsorption energy of −1.3 eV compared with −0.87 and −0.03 eV, respectively. In Figure 2d–f, charge density difference maps also reveal more extensive electron redistribution at the TEA/Ti_3_C_2_T_x_/Co-BDC interface, which confirmed a stronger charge transfer from TEA to the sensing material.

Liu et al. [63] subsequently prepared SnO_2_/TiO_2_ heterostructures (MST-X) by calcining Sn-MOF/Ti_3_C_2_T_x_ precursors. MXene facilitated Sn-MOF growth and was converted into TiO_2_, forming closely coupled n–n SnO_2_/TiO_2_ interfaces. The optimized MST-2-based gas sensor achieved a high response of 3525.2 at 10 ppm TEA at 61 °C. This behavior of the prepared gas sensing material was attributed to its mesoporous structure, enlarged surface area, oxygen-vacancy-rich surface, and interfacial electron transfer from TiO_2_ to SnO_2_, which promoted oxygen activation and TEA oxidation. However, the depth mechanism and reproducibility for this high response should be further evaluated for its practical application.

### 2.2. Doped and Surface-Modified MOF-Based Materials

Tian et al. [64] reported an iron-doped cobalt MOF (Fe/Co-MOF)-derived cobalt oxide (Co_3_O_4_) composite for TEA detection. The sensing materials were synthesized using a hydrothermal method followed by calcination using terephthalic acid as the organic ligand and Fe^3+^ as the dopant to regulate the local electronic structure of Co_3_O_4_. It was found that at 5%, Fe-doped Co_3_O_4_ showed the highest sensing performance, with a response of 21 at 100 ppm TEA at a temperature of 220 °C. The proposed gas sensor also exhibited a fast response/recovery time of 32/34 s, good repeatability, improved selectivity toward TEA, and an interesting detection limit of 5 ppm. The mechanistic analysis suggested that Fe doping promoted charge transfer, shifted the electronic structure, increased active adsorption sites, and optimized the interaction between TEA and the sensing surface. This study suggests that controlled Fe doping may be an effective strategy to improve the sensing activity, selectivity, and stability of Co-MOF-derived Co_3_O_4_-based TEA gas sensors. Shanmugam et al. [65] also investigated the structural engineering of Co-MOF pores using trisodium citrate as a surface-modifying and chelating agent for room-temperature TEA detection. The 5 mM trisodium citrate-treated sample (Co-MOF-2) showed a more porous dodecahedral morphology, higher surface area, and more oxygen-vacancy-related surface defects compared to the untreated Co-MOF. This structural tuning improved the accessibility of TEA molecules and strengthened surface interaction during sensing. The Co-MOF-2 sensor achieved a maximum responsivity of 144% at 400 ppm TEA at 25 °C, with faster response/recovery behavior, improved selectivity, and an experimental detection limit of 25 ppm and theoretical detection limit of 4 ppm. It was considered that enhanced response was attributed to the optimized porous framework, abundant surface area, and defect-rich surface chemistry. In our opinion, it is clear that the above-mentioned study provides a simple route for developing room-temperature MOF-based TEA sensors without relying on high operating temperatures.

### 2.3. MOF-Derived Fe_2_O_3_ Materials

**Figure 3:**
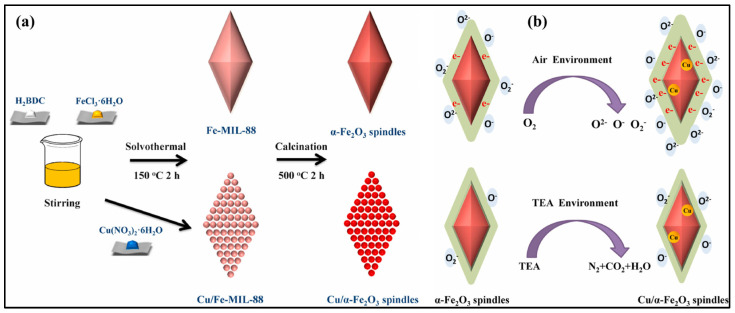
(**a**) Schematic diagram showing the formation of Cu-doped α-Fe_2_O_3_ porous spindles and (**b**) sensing mechanism for TEA detection. Reproduced with permission [66].

Gao et al. [66] developed copper-doped alpha iron oxide (Cu-doped α-Fe_2_O_3_) porous spindles using MIL-88-Fe as the precursor (Figure 3a). The spindle-like morphology of α-Fe_2_O_3_ was retained after calcination, whereas Cu incorporation restrained crystal growth, reduced grain size, increased surface area, and introduced more oxygen-vacancy-related active sites. X-ray photoelectron spectroscopy (XPS) analysis confirmed that Cu doping increased V_O_ and surface-chemisorbed oxygen contributions from 14.7% to 47.7%, supporting stronger oxygen adsorption and faster surface reactions. The 0.5 wt% Cu-doped α-Fe_2_O_3_ showed better TEA sensing behavior with a response of 31.7 at 100 ppm TEA at 240 °C. The TEA gas sensor also showed a rapid response/recovery behavior of 2/7 s. This study demonstrates that Cu doping may be an effective approach to improve the gas sensing behavior of the MOF-derived α-Fe_2_O_3_ by combining porous morphology with defect-mediated sensitization. Figure 3b illustrates the proposed TEA sensing mechanism of Cu-doped α-Fe_2_O_3_ porous spindles. In air, oxygen molecules adsorb on the α-Fe_2_O_3_ surface and capture electrons from the conduction band, forming reactive oxygen species such as O_2_^−^, O^−^, and O^2−^. This electron withdrawal forms an electron-depletion layer and increases sensor resistance. On introducing TEA, it diffuses through the porous spindle structure and reacts with the adsorbed oxygen species, producing N_2_, CO_2_, and H_2_O while releasing electrons back to α-Fe_2_O_3_. This electron return reduces the resistance and generates the sensing signal. Figure 3b also suggests the important role of Cu doping. Cu ions substitute Fe^3+^ sites and induce lattice defects and V_O_, which provide more active adsorption sites for oxygen molecules. These oxygen vacancies increase the amount of chemisorbed oxygen and accelerate the surface reaction with TEA.

Ma et al. [67] synthesized hollow ship-like alpha iron oxide (α-Fe_2_O_3_) with V_O_ defects using MIL-88A (Fe) as a MOF precursor followed by calcination. The obtained α-Fe_2_O_3_ retained hollow and porous microstructure composed of nanoparticles (NPs) with rough surfaces and thin shells, offering efficient gas diffusion channels and abundant adsorption sites. By optimizing the calcination temperature, the authors tuned the V_O_ concentration and morphology. It was observed that F600 sample exhibit better TEA sensing performance. At 180 °C, α-Fe_2_O_3_ based gas sensor exhibited response of 527 towards 50 ppm TEA and rapid response/recovery times of 20/8 s at 2 ppm. The sensor also displayed excellent selectivity towards TEA. The enhanced gas sensing performance was attributed to the hollow porous architecture, suitable oxygen vacancy concentration and improved surface reactivity. Zhao et al. [68] reported selenium modified MOF-derived spindle-shaped alpha iron oxide (α-Fe_2_O_3_) for enhanced TEA sensing. The α-Fe_2_O_3_ substrate was obtained from Fe-based MOF precursor followed by surface modification with different selenium (Se) contents to form Se/α-Fe_2_O_3_ Schottky structures. Under the optimized conditions, 10 wt% Se modified α-Fe_2_O_3_ sample showed the better gas sensing performance and delivered response of 20.8 towards 100 ppm TEA at 275 °C. In addition, above mentioned gas sensor also exhibited a fast response time of ≤4 s, recovery time of 94 s, detection limit of 0.2 ppm, decent TEA selectivity, and good humidity stability. The improvement in the TEA detection was ascribed to the formation of Schottky junction between Se and α-Fe_2_O_3_, introduction of new adsorption sites, increased V_O_ and chemisorbed oxygen contents.

### 2.4. MOF-Derived Co_3_O_4_ Materials

Sun et al. [69] synthesized gallium-doped cobalt oxide (Ga-doped Co_3_O_4_) hierarchical bow-like architectures using cobalt MOF precursors. The structures were assembled from ultrathin porous nanosheets, providing a large surface area and abundant diffusion channels for TEA molecules. The 2 at % Ga-doped Co_3_O_4_ showed better sensing performance with high response of 108 toward 50 ppm TEA at 180 °C. This gas sensor also showed rapid response/recovery times of 3/15 s, detection limit of 0.1 ppm, excellent selectivity, repeatability, and long-term stability. The enhancement was attributed to Ga-induced electronic modulation, increased oxygen species, improved catalytic activity, and the porous hierarchical morphology inherited from the MOF precursor. Du et al. [70] reported MOF-derived molybdenum-doped cobalt oxide (Mo-doped Co_3_O_4_) hierarchical flower-like structures for TEA sensing. The materials were obtained through a solvothermal process followed by annealing approach, where Co-MOF acted as the structural precursor and Mo was introduced to tune the surface and electronic properties of Co_3_O_4_. The optimized 1 at % Mo-doped Co_3_O_4_ sensor exhibited decent response of 92 toward 50 ppm TEA at 180 °C which was about 46 times higher than pure Co_3_O_4_. It also showed short recovery time of 33 s, good selectivity, repeatability, and long-term stability. The improved performance was mainly related to the combined effect of Mo doping and the hierarchical flower-like microstructure, which offered more active sites, better gas diffusion, and enhanced surface reaction activity. Chen et al. [71] developed Co_3_O_4_ based gas sensor by directly growing Co-MOF array films on inter-digital electrode substrates followed by annealing to convert the films into Co_3_O_4_. Unlike conventional powder-coating methods, this approach produced uniform, well-contacted sensing films with controllable thickness and open array structures which improved conducting pathways and gas accessibility. The optimized Co_3_O_4_-P-4 based gas sensor showed excellent response of 230 towards TEA at 200 °C. It also showed much faster response behavior, decreasing from 82 s to 9 s, along with improved selectivity and anti-humidity performance. The enhanced sensing behavior was attributed to abundant oxygen vacancies, open-space array morphology, and better electrical contact between the sensing film and electrodes. Sun et al. [72] synthesized aluminum/molybdenum co-doped Co_3_O_4_ (Al/Mo-Co_3_O_4_) porous hollow tetrahedrons using a Co-MOF precursor followed by annealing. The obtained three-dimensional (3D) hollow porous structure offers larger surface area, high gas permeability, and reduced particle aggregation whereas Al and Mo co-doping tuned the electronic structure and promoted surface reaction activity. The optimized 0.2 at % Al/Mo-Co_3_O_4_ based gas sensor delivered response of 132 toward 100 ppm TEA at 160 °C. It also showed fast response/recovery times of 4/36 s, detection limit of 0.5 ppm, good selectivity, and long-term stability. The improved performance was attributed to the synergistic effects of the hollow porous tetrahedral structure and Al/Mo co-doping. Sun et al. [73] reported one-dimensional (1D) ruthenium/molybdenum co-doped cobalt oxide (Ru/Mo-Co_3_O_4_) hollow microtubes derived from MOF precursors for TEA detection. The 1D hollow architecture provided diffusion channels, reduced aggregation, and increased accessible surface sites whereas Ru and Mo co-doping further improved catalytic activity and surface oxygen interaction. The 0.3% Ru/Mo-Co_3_O_4_ showed interesting gas sensing performance, exhibiting high response of 126 towards 100 ppm TEA at 160 °C. This gas sensor also exhibited very fast response/recovery times of 5/7 s, good selectivity, repeatability, and long-term stability. Ding et al. [74] synthesized Fe-Co_3_O_4_ with coral flower-like structure using Co-MOF-74 as a precursor. Different Fe doping were investigated, and 2 mol % Fe-Co_3_O_4_ sample showed best gas sensing performance. This sensor delivered response of 21.2 toward 100 ppm TEA about five times higher than pure Fe-Co_3_O_4_ with short response/recovery times of 13/15 s. This gas sensor also showed a detection limit of 1 ppm along with acceptable selectivity and humidity resistance. The enhanced performance may be linked to the increased Co^2+^/Co^3+^ ratio, higher V_O_ content, more active sites for TEA reaction, and formation of a p-n hetero-junction between Co_3_O_4_ and Fe_2_O_3_ which accelerated adsorption-desorption kinetics.

### 2.5. Fe, Zn, and In-Based Mixed-Oxide Materials

**Figure 4:**
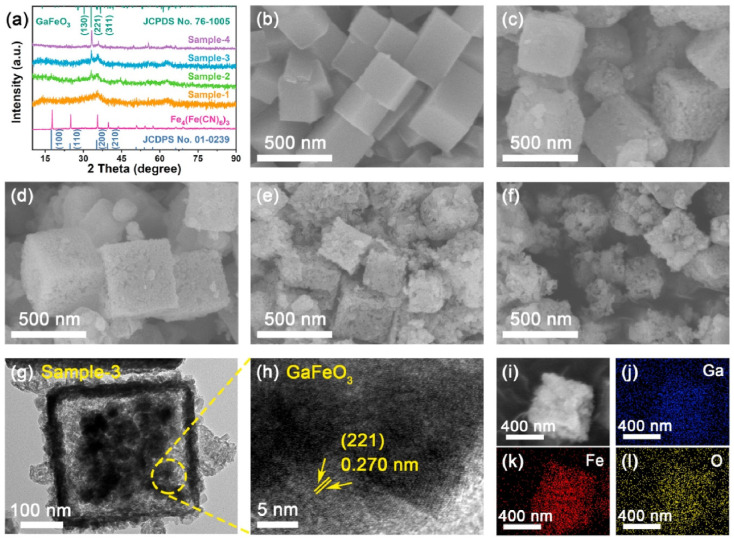
(**a**) Simulated and experimental XRD patterns of the Fe_4_[Fe(CN)_6_]_3_ MOFs (JCPDS No. 01-0239) and experimental XRD patterns of Samples -1, -2, -3, -4 and the simulated XRD pattern of GaFeO_3_ phase (JCPDS No. 76–1005). Field-emission scanning electron microscopy (FE-SEM) images of (**b**) Fe_4_[Fe(CN)_6_]_3_ MOF precursor, and (**c**–**f**) GaFeO_3_ microcubes of samples -1, -2, -3 and -4 obtained at 400, 450, 500 and 550 °C, respectively. (**g**) TEM and (**h**) HRTEM picture of sample-3. (**i**) Selected SEM picture of sample-3 and corresponding energy-dispersive X-ray spectroscopy (EDX) mapping pictures for (**j**) Ga, (**k**) Fe and (**l**) O elements, respectively. Reproduced with permission [78].

Liu et al. [75] prepared a fern-like indium oxide/zinc oxide (In_2_O_3_/ZnO) composite using MIL-68/zeolitic imidazolate framework-8 (ZIF-8) MOF precursors as sacrificial templates for TEA sensing. The optimized In_0.3_Zn sample showed a distinctive morphology in which thin ZnO/In_2_O_3_ nanowires grew on porous microrods, forming mesoporous fern-like architecture with abundant gas diffusion pathways. This structure may provide large number of exposed active sites and support the interactions between TEA molecules and surface-adsorbed oxygen species. The In_0.3_Zn sensor exhibited a response of 44.6 towards 100 ppm TEA at temperature of 100 °C with response/recovery times of 14/36 s. It also detected TEA down to 10 ppm and showed a high response of 171.6 at 1000 ppm. The enhanced sensing behavior was attributed to the mesoporous structure, high surface area, abundant nanowire-on-microrod architecture, and n-n heterojunction formation between In_2_O_3_ and ZnO. Li et al. [76] prepared hierarchical bimetallic MOF-derived zinc ZnO/NiO (ZnO/NiO) composite. The Ni^2+^ was introduced into the Zn-based MOF precursor, and subsequent calcination formed flower-like ZnO/NiO composites with abundant mesopores, high surface area, and well-connected p-n hetero-junctions. The optimized ZnO/NiO sensor showed a response of 49.8 towards TEA at 200 °C with response time of 11 s, good anti-interference ability, and long-term stability for at least 70 days. The improved TEA sensing performance was attributed to the reduced band gap, high specific surface area, rich porosity, increased oxygen vacancies, and efficient charge transfer across the ZnO/NiO interface. Li et al. [77] developed hierarchical kiwifruit-like zinc oxide/zinc ferrite (ZnO/ZnFe_2_O_4_) hetero-structures using iron-doped Zn-based MOF precursors followed by thermal decomposition. The obtained material possessed nanosheet-like surface assembled from interpenetrated ZnO and ZnFe_2_O_4_ NPs, forming porous hierarchical architecture which promoted gas diffusion and surface reaction with TEA molecules. Compared with single-component ZnO, ZnO/ZnFe_2_O_4_ hetero-structure showed improved TEA sensing performance and exhibited interesting response of 40.5 at 200 °C. It also exhibited response/recovery times of 32/41 s and maintained decent stability for 1 month. The enhanced sensing behavior was linked to the kiwifruit-like morphology, accessible reactive sites, and hetero-junction-driven interfacial charge transfer. Yu et al. [78] prepared hierarchical hollow gallium ferrite (GaFeO_3_) microcubes through a Ga^3+^ modified Fe-based Prussian blue (PB) MOF template conversion strategy. By adjusting the calcination temperature, the authors controlled the hollow structure, particle assembly, and gas-accessible morphology of the GaFeO_3_ microcubes. The sample calcined at 500 °C showed the higher TEA sensing behavior and exhibited response of 7.4 toward 200 ppm TEA at 200 °C with rapid response/recovery times of 9/49 s. The sensor also displayed good selectivity. Although the response value was lower than some other MOF-derived oxide sensors, the work is important because it introduces GaFeO_3_ as a hollow multi-metal oxide sensing material with stable and selective TEA response. The enhanced performance was attributed to the loose hollow interior, NPs assembled architecture, and improved permeability for TEA diffusion. Figure 4 presents the structural and morphological confirmation of the synthesized hollow GaFeO_3_ microcubes. In Figure 4a, the X-ray diffraction (XRD) pattern of the Fe_4_[Fe(CN)_6_]_3_ PB precursor shows sharp peaks at 17.37°, 24.71°, 35.16°, and 39.49°, suggesting its high crystallinity and purity. After Ga^3+^ modification and calcination, Samples 1–4 show diffraction peaks matching orthorhombic GaFeO_3_, especially at 30.28°, 33.04°, and 36.76°, corresponding to the (130), (221), and (311) planes. The peaks become sharper as the annealing temperature increases from 400 to 550 °C, indicating improved crystallinity. The scanning electron microscopy (SEM) images in Figure 4b-f shows that the original PB precursor has a smooth cubic morphology with a size of about 0.3–0.5 μm whereas the calcined GaFeO_3_ samples largely retained this cubic shape. However, their surface structures change with calcination temperature. Samples 1 and 2 show rough microcubes with tightly stacked NPs whereas Figure 4e shows that Sample 3, prepared at 500 °C, consists of loosely aggregated GaFeO_3_ NPs. This loose and porous structure is important because it can provide more accessible surface sites and easier gas diffusion pathways for TEA sensing. The transmission electron microscopy (TEM) image in Figure 4g further confirms that Sample 3 retained cubic morphology but develops a clear hollow interior after pyrolysis. In Figure 4h, high-resolution transmission electron microscopy (HRTEM) image shows a lattice spacing of 0.270 nm, assigned to the (221) plane of GaFeO_3_, suggesting the crystalline nature of the prepared material. The elemental mapping images in Figure 4i–l show uniform distribution of Ga, Fe, and O throughout the microcube, supporting the successful formation of homogeneous GaFeO_3_.

Yu et al. [79] prepared In_2_O_3_-NiO hierarchical hollow spheres using a Ni-MOF precursor followed by cation exchange with In^3+^ and calcination. This cation-exchange strategy helped preserve the porous reticular skeleton of the original MOF during thermal conversion, producing hollow spheres assembled from nanosheets. The optimized In_2_O_3_-NiO based gas sensor showed response of 33.9 toward 100 ppm TEA at 200 °C with acceptable selectivity, long-term stability, and a low detection limit of 500 ppb. The high surface area of 55.5 m^2^/g and mesoporous hollow structure provided efficient diffusion pathways and abundant active sites for TEA adsorption. In addition, the p-n hetero-junction between n-type In_2_O_3_ and p-type NiO promoted interfacial charge transfer and improved the sensing response. Wang et al. [80] reported the fabrication of ZIF-8 derived neodymium oxide-decorated ZnO (Nd_2_O_3_-ZnO) nanocages for the quantification of TEA. The ZnO nanocage structure provided a porous framework whereas Nd_2_O_3_ nanorods improved oxygen adsorption and activation on the composite surface. The XPS and oxygen temperature-programmed desorption (O_2_-TPD) confirmed increased chemisorbed oxygen species in the prepared Nd_2_O_3_-ZnO composite. The Nd_2_O_3_-ZnO based gas sensor showed a response 15.7 times higher than pure ZnO towards 100 ppm TEA with decent detection limit of 150 ppb. The enhanced sensing behavior was attributed to the porous ZnO nanocage structure, Nd_2_O_3_ assisted oxygen activation, and hetero-junction-driven electronic modulation. Zhai et al. [81] synthesized nickel/iron-based bimetallic MOF-derived nickel ferrite (NiFe_2_O_4_, NFO) polyhedrons for TEA sensing. By changing the solvent composition, the authors obtained larger NFO polyhedrons with improved morphological and structural stability compared with the smaller-sized products. The large NFO polyhedron-based sensor showed a response of 18.9 toward 50 ppm TEA at 190 °C with fast response time of 6 s, good selectivity, and repeatability. The performance was attributed to the bimetallic MOF-derived polyhedral structure which offered accessible surface sites and favorable gas diffusion pathways. In another study, Zhai et al. [82] synthesized porous MOF-based ZnO/zinc ferrite (ZnO/ZnFe_2_O_4_, ZZFO) structures using a PB analogue as a self-sacrificial template. The obtained material consisted of homogeneous porous structures built from abundant primary nanocrystallites providing fast gas access and surface reaction pathways. The ZZFO sensor showed excellent TEA sensing performance, especially ultrafast response and recovery times of about 1/9 s towards 100 ppm TEA at 170 °C. It also showed relatively low operating temperature and attractive long-term stability.

### 2.6. Ni, V, and Zr Based Mixed Oxides

Geng et al. [83] developed bimetallic nickel/vanadium MOF-derived nickel vanadate/NiO (Ni_3_V_2_O_8_@NiO = NV@NiO) hollow microspheres for sensitive TEA detection. The materials were synthesized through solvothermal process, cation-exchange treatment, and annealing which formed hollow p-p hetero-structured microspheres with high porosity and large surface area. The optimized NV@NiO-2 sensor showed a response of 43.7 towards 100 ppm TEA at 240 °C with response/recovery times of 88/127 s. This gas sensor also achieved detection limit of 4.5 ppb and showed linearity over 1–100 ppm TEA. The improved sensing behavior was attributed to the hollow architecture, porous diffusion channels, p-p hetero-junction between Ni_3_V_2_O_8_ and NiO, and enhanced surface reaction activity. Geng et al. [84] synthesized NiO/zirconium dioxide (NiO/ZrO_2_) hollow microspheres from bimetallic Ni/Zr-MOF precursors. The annealed composites formed p-p hetero-structures with hollow spherical morphology, high porosity, and accessible gas diffusion pathways. The optimized NiO/ZrO_2_-2 sensor showed a response of 32.3 towards 100 ppm TEA at 240 °C with response/recovery times of 55/83 s and detection limit of 7.2 ppb. It also showed excellent repeatability and a strong linear relationship between response and TEA concentration from 1 to 200 ppm. The improved performance was attributed to the hollow MOF-derived structure, large active interface, and p-p hetero-junction between NiO and ZrO_2_. Wang et al. [85] developed bimetallic MOF-derived NiO/SnO_2_ (NiO-SnO_2_) nanomaterials for highly sensitive TEA detection. The sensing materials were prepared by solvothermal method using Sn and Ni precursors followed by calcination to form the NiO-SnO_2_ composites with p-n hetero-junctions. The optimized 0.5 mol % NiO-SnO_2_ sensor exhibited the decent response of 124.5 towards 50 ppm TEA at 170 °C which was 1.5 times higher than the SnO_2_ sensor. The sensor showed decent selectivity towards TEA with responses 3.1–49.8 times higher than those for other tested gases. The improved sensing performance was attributed to increased surface area, abundant oxygen vacancies, reduced band gap, and interfacial charge modulation at the NiO/SnO_2_ p-n hetero-junction.

### 2.7. MOF-Derived Oxide/Carbon Composites

**Figure 5:**
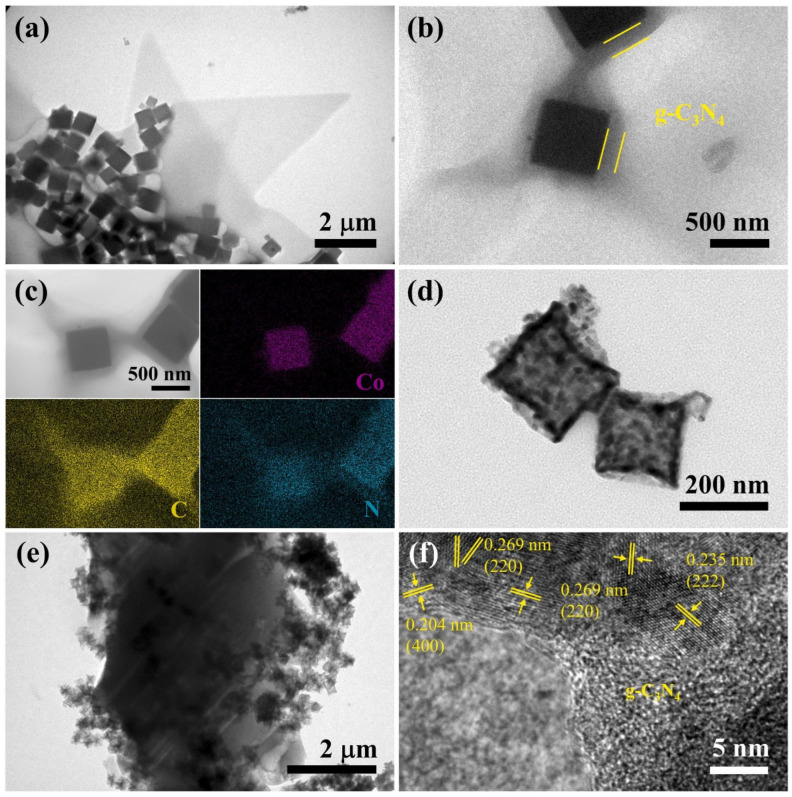
(**a**,**b**) TEM, (**c**) scanning TEM and elemental mapping pictures of g-C_3_N_4_ coupled ZIF-67. TEM pictures of (**d**) Co_3_O_4_, and (**e**) CNCO-2. (**f**) HRTEM image of CNCO-2. Reproduced with permission [87].

Wei et al. [86] prepared MOF-derived copper/carbon-modified zinc oxide (Cu/C-ZnO) nanosheets for TEA detection using ZIF-8-derived strategy. The material was obtained through solution-based Cu introduction followed by two-step calcination which preserved the nanosheet morphology whereas introducing an in situ carbon phase and highly dispersed Cu^2+^ clusters. The optimized Cu/C-ZnO composite showed response of 225 towards 100 ppm TEA at 280 °C which was 2.18 times higher than pure ZnO and 1.22 times higher than carbon modified ZnO (C-ZnO). It also showed faster response/recovery kinetics of 17/38 s. The sensing enhancement was attributed to the hierarchical nanosheet network, conductive carbon phase, Cu-related hetero-junction-like interface, and increased V_O_ content of 30.6%. Wang et al. [87] prepared graphitic carbon nitride coupled Co_3_O_4_ (g-C_3_N_4_/Co_3_O_4_) hollow nanocubes using ZIF-67 derived Co_3_O_4_ as the base material for TEA detection. The surface morphology plays vital role in gas sensing applications. In Figure 5a,b, TEM images reveal that ZIF-67 nanocubes were successfully assembled on two-dimensional (2D) g-C_3_N_4_ nanosheets which is also suggesting close contact between the MOF precursor and g-C_3_N_4_. Figure 5c further supports this coupling through scanning TEM and elemental mapping, where Co is mainly distributed in the nanocube region whereas C and N signals correspond to the g-C_3_N_4_ sheet which is confirming the coexistence and surface coating structure. After calcination, Figure 5d shows that pristine Co_3_O_4_ retained the cubic morphology of ZIF-67 but develops a distinct hollow nanocube structure. In Figure 5e, optimized g-C_3_N_4_/Co_3_O_4_ (CNCO-2) composite clearly show Co_3_O_4_ hollow nanocubes distributed on g-C_3_N_4_ nanosheets, indicating that the hybrid structure is preserved after thermal conversion. The HRTEM image in Figure 5f shows lattice spacing of about 0.269, 0.235, and 0.204 nm, assigned to the (220), (222), and (400) planes of cubic Co_3_O_4_, respectively. The regions without clear lattice fringes are attributed to low-crystallinity g-C_3_N_4_ sheets. In addition, coupling of 2D g-C_3_N_4_ with Co_3_O_4_ regulated the surface V_O_ and chemisorbed oxygen (OC) which plays vital role for gas sensing reactions. The fabricated composite based gas sensor showed higher response, faster response speed, and better selectivity towards TEA compared to the pristine Co_3_O_4_. The improvement in the gas sensing performance was attributed to oxygen-defect regulation, hetero-junction formation, Co d-band center modulation, and improved gas diffusion through the coupled structure.

### 2.8. MOF-Derived In_2_O_3_ Based Materials

Liu et al. [88] reported porous indium oxide (In_2_O_3_) microtubes derived from MIL-68(In) MOF precursors for sub-ppm TEA detection. The MOF precursor was converted into hollow In_2_O_3_ microtubes through calcination while largely preserving the original rod-like morphology and forming porous structure. The obtained microtubes were approximately 7–9 μm long and 0.8–1.1 μm in diameter, providing efficient gas diffusion channels and abundant surface defects. The sensor exhibited high response of 145 toward 1 ppm TEA at 140 °C with a short response time of 5 s and recovery time of 20 s. It also showed good reversibility, excellent selectivity, and a detection limit as low as 100 ppb. Sun et al. [89] synthesized porous lacunaris indium oxide (In_2_O_3_) from In-MIL-68 through annealing for ppb-level TEA detection. The optimized In_2_O_3_ obtained at 500 °C showed a highly porous structure that enabled efficient gas diffusion and abundant surface reaction sites. The sensor achieved a response of 32 toward 100 ppb TEA at 120 °C with response/recovery times of 9/36 s. It also showed good linearity over 0.1–5 ppm TEA, excellent selectivity, 40-day stability, and strong moisture resistance, retaining 86.2% of its response at 90% relative humidity. Miao et al. [90] reported V_O_-rich indium oxide (In_2_O_3_) hollow prism-like nanoflowers derived from amino-functionalized MIL-68(In) [NH_2_-MIL-68(In)]. By controlling the annealing temperature, the authors optimized the V_O_ concentration, surface area, and electron mobility of the In_2_O_3_ material. The In_2_O_3_-400 sample, prepared at 400 °C, showed the improved sensing performance and delivered response of 684.32 towards 7 ppm TEA at 100 °C. Remarkably, the sensor achieved good detection limit of 0.046 ppb with excellent selectivity, repeatability, and long-term stability. This enhanced performance was attributed to the hollow mesoporous prism-like nanoflower structure, more V_O_, large surface area, and improved electron transport. Han et al. [91] engineered MOF-derived In_2_O_3_ with different V_O_ contents and crystalline phases by pyrolyzing NH_2_-MIL-68(In) at different temperatures. The obtained In_2_O_3_ at 400 °C contained mixed hexagonal and cubic phases, abundant oxygen vacancies, and enlarged surface/interface area. This MOF-In_2_O_3_-400 material acted as a dual-functional gas sensor and showed temperature-dependent selectivity towards nitrogen dioxide (NO_2_) and TEA.

### 2.9. MOF-Derived In_2_O_3_-Based Materials

**Figure 6:**
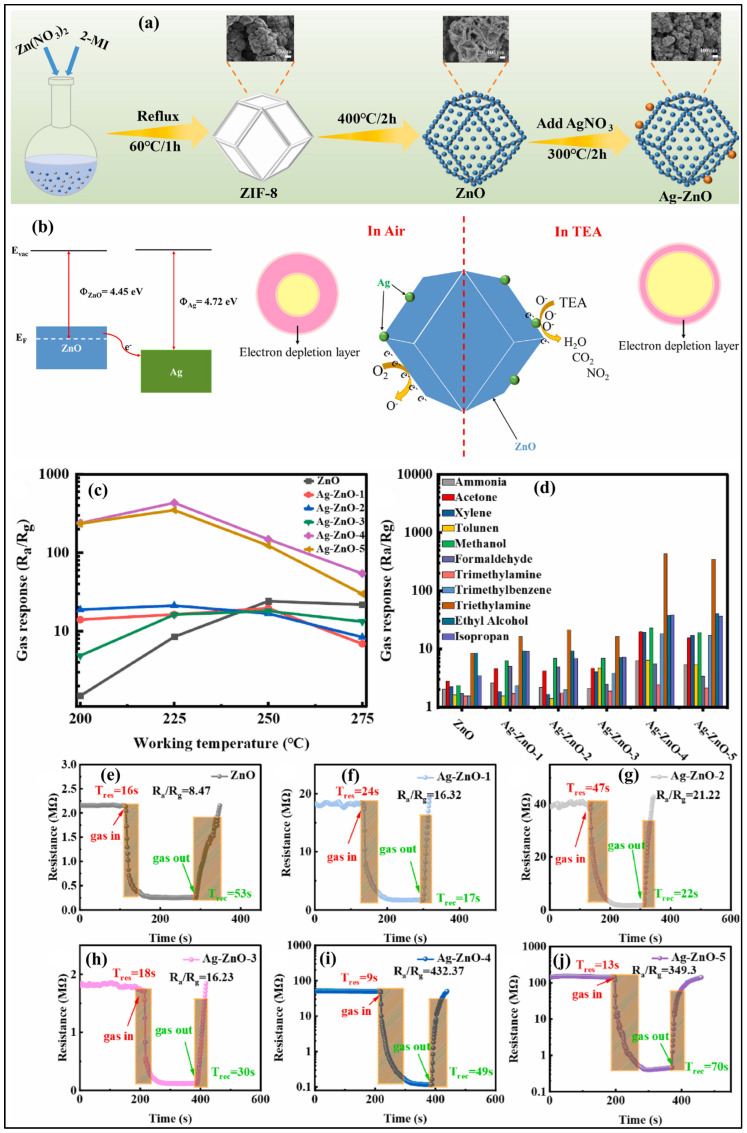
(**a**) Schematic illustration of the preparation of Ag-ZnO. (**b**) Sensing mechanism for TEA detection. (**c**) Responses of pristine ZnO and different Ag-ZnO based gas sensors for 100 ppm TEA under different temperatures and (**d**) cross responses of the ZnO and different Ag-ZnO exposed in various gases. (**e**–**j**) Response transient data of ZnO and different Ag-ZnO based sensors for 100 ppm TEA at 225 °C. Reproduced with permission [92].

Li et al. [92] designed silver (Ag) NPs decorated MOF-derived zinc oxide (Ag NP/ZnO) NPs for rapid and highly responsive TEA vapor detection. MOF-derived ZnO was prepared from ZIF-type precursor, followed by Ag NPs decoration with different Ag contents (Figure 6a). The optimized 1.3 mol % Ag NP/ZnO sample (Ag-ZnO-4) showed interesting sensing performance, achieving high response of 430.6 towards 100 ppm TEA at 225 °C. The sensor also detected TEA to 1 ppm and exhibited short response/recovery times of 9/49 s, good selectivity, reproducibility, and stability of 30 days. It was considered that improved gas sensing performance of this TEA sensor may be attributed to the catalytic effect of Ag NPs, increased surface area, enriched V_O_, and more active sites for TEA adsorption and oxidation. The sensing mechanism for TEA monitoring has been described in Figure 6b. As shown in Figure 6c, pristine ZnO reaches its maximum response at 250 °C, whereas Ag-ZnO-4 exhibits significantly higher response of approximately 430 toward 100 ppm TEA at a lower operating temperature of 225 °C. Excessive Ag loading in Ag-ZnO-5 slightly decreases the response, probably because Ag NPs block active surface sites. Figure 6d confirms the superior selectivity of Ag-ZnO-4 toward TEA, with a response of approximately 430, compared with about 50 for ethanol and isopropanol and below 50 for the other interfering gases. The dynamic curves in Figure 6e–j show response/recovery times of 16/53, 24/17, 47/22, 18/30, 9/49, and 13/70 s for ZnO, Ag-ZnO-1, Ag-ZnO-2, Ag-ZnO-3, Ag-ZnO-4, and Ag-ZnO-5, respectively. Among them, Ag-ZnO-4 provides the highest response and fastest response time, demonstrating that an optimized Ag content accelerates TEA adsorption and surface reaction, although higher Ag loading prolongs recovery.

**Figure 7:**
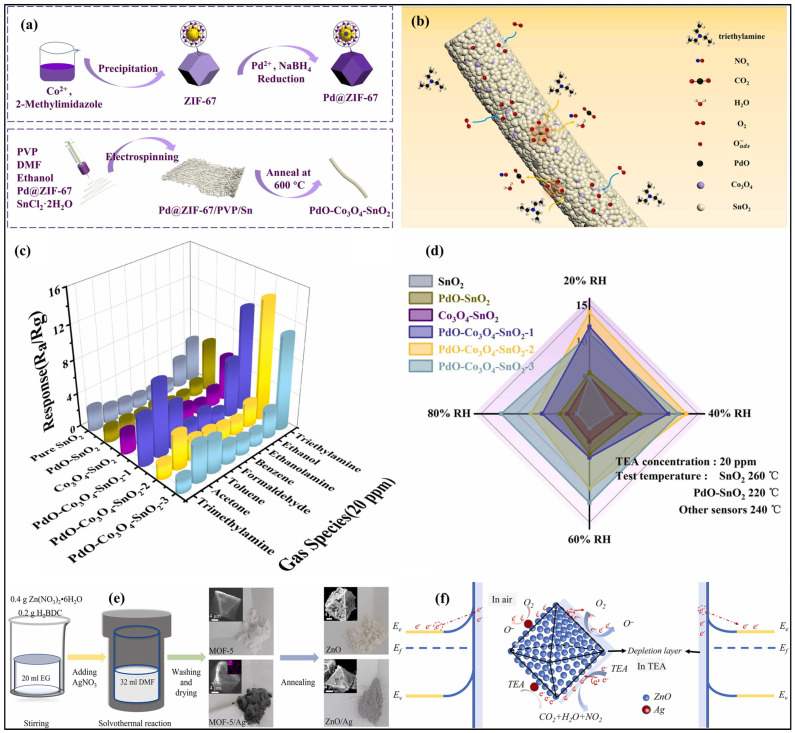
(**a**) Schematic diagram for the synthesis of sensing material. (**b**) Possible sensing mechanism for TEA detection using PdO-Co_3_O_4_-SnO_2_ composite. (**c**) Selectivity of various fabricated TEA gas sensors. (**d**) Effect of humidity on different TEA gas sensors. (**e**) Schematic illustration for the preparation of 3D ZnO and ZnO/Ag micro-octahedra and (**f**) gas sensing mechanism. Reproduced with permission [94,97].

Liu et al. [93] prepared mesoporous ruthenium-doped SnO_2_ (Ru-SnO_2_) from bimetallic Sn/Ru MOF for TEA sensing under high humidity. The one-step MOF-derived synthesis formed the porous SnO_2_ based material with a high specific surface area of 69.48 m^2^/g whereas Ru^3+^ substitution helped tune carrier concentration and improve surface reactivity. Among the prepared samples, 0.4 mol % Ru-SnMOF@SnO_2_ exhibited better TEA sensing performance at 250 °C with high sensitivity, fast response kinetics, good selectivity, and long-term stability. In addition, the proposed gas sensor retained a strong response of 125.5 toward 100 ppm TEA at 80% relative humidity making it especially relevant for humid environments such as seafood freshness monitoring. The enhancement was attributed to the mesoporous structure, Ru-induced electronic modulation, and improved gas capture ability. In another study, Wang et al. [94] prepared PdO and Co-MOF derivative modified SnO_2_ (PdO-Co_3_O_4_-SnO_2_) nanofibers for rapid TEA detection (Figure 7a). The Pd NPs were confined in ZIF-67 and then converted into PdO and Co_3_O_4_ NPs uniformly decorated on SnO_2_ nanofibers through electrospinning and calcination. The optimized 0.012 wt% PdO-Co_3_O_4_-SnO_2_ based gas sensor showed response of 14 towards 20 ppm TEA at 240 °C with fast response time of 3 s and detection limit of 1 ppm. The improved sensing performance was attributed to V_O_, p-n junctions, PdO electronic sensitization, and the 1D nanofiber morphology. The sensing mechanism for TEA detection has been illustrated in Figure 7b. The sensor also showed excellent selectivity (Figure 7c) humidity resistance (Figure 7d) because PdO inhibited hydroxyl poisoning while Co_3_O_4_ assisted oxidation reactions. As illustrated in Figure 7b, enhanced TEA response of the PdO-Co_3_O_4_-SnO_2_ nanofibers arises from the combined effects of surface oxygen chemistry, heterojunction formation, and PdO mediated catalysis. In air, oxygen molecules capture electrons from n-type SnO_2_ and are converted mainly into O^−^ species at 240 °C, producing an electron-depletion layer and increasing the sensor resistance. Upon exposure to TEA, adsorbed molecules react with these oxygen species to form CO_2_, H_2_O, and nitrogen-containing oxidation products, while the released electrons return to the SnO_2_ conduction band and sharply decrease the resistance. The substitution of Sn^4+^ by Co^2+^/Co^3+^ generates additional oxygen vacancies, providing more sites for oxygen and TEA adsorption. Meanwhile, p-type PdO and Co_3_O_4_ form p-n heterojunctions with SnO_2_, forming wider depletion regions and amplifying the resistance change during gas exposure. PdO further acts as an electronic and catalytic sensitizer: it is partially reduced from Pd^2+^ to Pd^0^ in TEA, facilitating electron transfer and lowering the activation barrier for TEA oxidation, before being reoxidized in air. The porous, NPs assembled 1D nanofibers also promote gas diffusion, expose abundant reaction sites, and provide efficient axial electron-transport pathways. The reactions for the possible sensing mechanism towards the quantification of TEA can be explained as below.

(C_2_H_5_)_3_N(gas) → (C_2_H_5_)_3_N(ads) (1)

2(C_2_H_5_)_3_N(ads) + (39 + 2x)O^−^ (ads) → 2NO_x_ + 12CO_2_ + 15H_2_O + (39 + 2x)e^−^ (2)

(3) $${\mathrm{Co}}_{3}O_{4}\;\overset{{\mathrm{SnO}}_{2}}{\to }\;{{\mathrm{Co}}^{\prime \prime }}_{\mathrm{Sn}}\;+\;2{{\mathrm{Co}}^{\prime }}_{\mathrm{Sn}}\;+\;4O_{0}^{x}+2v_{\ddot{O}}$$

Guo et al. [95] developed PdO/ZnO/In_2_O_3_ nanofibers using Pd@ZIF-8 as a MOF templated catalyst for TEA sensing. The Pd NPs confined in ZIF-8 were transformed into ultra-small PdO NPs in the composite nanofibers, allowing efficient catalytic sensitization with only 0.2 mol% PdO. Compared with pure In_2_O_3_ and ZnO-In_2_O_3_ nanofibers, PdO-ZnO-In_2_O_3_ showed better TEA sensing behavior with a response of 386 towards 100 ppm TEA at 250 °C. The sensor also displayed good selectivity and a very fast response time of 1 s, although the recovery time was relatively long at 740 s. The enhancement was attributed to ultra-small PdO induced depletion effects and hetero-junctions between PdO/ZnO and In_2_O_3_. Guo et al. [96] fabricated palladium/palladium oxide-functionalized zinc oxide nanorods (Pd/PdO@ZnO-ZnO) using MOF templated catalyst strategy for selective TEA detection. In this design, Pd@ZIF-8 was used to generate ultra-small Pd and PdO species on ZnO nanorods. The Pd/PdO@ZnO-ZnO composite based gas sensor showed high response of 258 towards 50 ppm TEA at 275 °C. The enhanced response may be attributed to the catalytic and spillover effects of Pd/PdO, modulation of the sensor base resistance, and improved surface reaction activity. Sun et al. [97] prepared 3D ZnO/Ag micro-octahedra derived from MOF-5 (Figure 7e). The MOF-5 precursor decorated with Ag particles was calcined to form the hierarchical ZnO/Ag micro-octahedra assembled from ZnO nanosheets. This fabricated gas sensor exhibited response of 293.8 towards 10 ppm TEA at 200 °C with good selectivity and long-term stability. The improved sensing performance of this sensor was attributed to the hierarchical three-dimensional morphology, oxygen vacancies, and the catalytic spillover effect of Ag which promoted oxygen activation and TEA oxidation on the ZnO surface. The sensing mechanism has been illustrated in Figure 7f.

In brief, it can be stated that TEA sensing mechanism of MOF-5 derived 3D ZnO/Ag micro-octahedra involves surface oxygen adsorption, electron transfer, Ag-assisted catalytic spillover, and Mott-Schottky junction formation. In air, oxygen molecules are adsorbed on the ZnO surface and capture electrons from the conduction band, generating ionized oxygen species such as O_2_^−^, O^−^, and O^2−^. This electron removal forms an electron depletion layer on the n-type ZnO surface, bends the energy band upward, and increases the sensor resistance. When the sensor was exposed to TEA, TEA molecules react with the adsorbed oxygen species and release electrons back into the ZnO conduction band. As a result, the depletion layer becomes thinner, the band bending was reduced, and the resistance decreases. Since the sensor operates optimally at 200 °C, O^−^ was considered the dominant reactive oxygen species. Therefore, the main TEA oxidation pathway involves the reaction of adsorbed TEA with O^−^ to produce CO_2_, H_2_O, and NO_2_ whereas releasing electrons which accounts for the high response of the ZnO/Ag sensor. The incorporation of Ag further enhanced the sensing behavior through two major effects. First, Ag promotes the formation of oxygen vacancies and increases active oxygen adsorption sites, which accelerates the surface redox reaction. Second, because Ag and ZnO have different Fermi levels, a Mott-Schottky junction forms at the Ag/ZnO interface. Electrons transfer from ZnO to Ag, increasing the initial depletion layer and raising the air resistance. Upon TEA exposure, the catalytic spillover effect of Ag facilitates TEA oxidation and electron return to ZnO, causing a larger resistance change. Therefore, the improved TEA sensing performance of 3D ZnO/Ag micro-octahedra originates from the synergistic contribution of V_O_, Ag catalytic spillover, larger surface area, and interfacial Mott-Schottky modulation. The reactions involves in the sensing mechanism of TEA can be explained as below,

O_2_ (gas) → O_2_ (ads) (4)

O_2_(ads) + e^−^ → O^−^_2_ (ads)  (T ≪ 100 °C) (5)

O^−^_2_ (ads) + e^−^ → 2O^−^(ads)  (100 °C < T ≪ 300 °C) (6)

2O^−^(ads) + e^−^ → O^2−^(ads)  (T > 300 °C) (7)

N(C_2_H_5_)_3_(gas) → N(C_2_H_5_)_3_(ads) (8)

2N(C_2_H_5_)_3_(ads) + 43/2O_2_^−^ → 2NO_2_ + 15H_2_O + 12CO_2_ + 43/2e^−^  (T ≪ 100 °C) (9)

2N(C_2_H_5_)_3_(ads) + 43O^−^ → 2NO_2_ + 15H_2_O + 12CO_2_ + 43e^−^ (100 °C < T ≪ 300 °C) (10)

2N(C_2_H_5_)_3_(ads) + 43O^2−^ → 2NO_2_ + 15H_2_O + 12CO_2_ + 86e^−^  (T > 300 °C) (11)

He et al. [98] developed gold-loaded indium oxide (Au/In_2_O_3_) hollow hexagonal prisms derived from MIL-68(In) for sensitive TEA detection. The MIL-68(In)-derived hollow prism structure provided a large surface area and abundant active sites, whereas Au decoration enhanced oxygen adsorption, oxygen dissociation, and charge modulation. Among the prepared samples, 0.6% Au/In_2_O_3_ showed the improved performance and delivered response of 476.3 towards 100 ppm TEA at 200 °C. The proposed sensor also showed good stability, with deviation below 5.6%, and decent selectivity with a TEA/ethanol response ratio of 28.8. The improved sensing behavior was mainly attributed to the hollow hexagonal morphology and Au-induced catalytic sensitization which together accelerated gas diffusion and surface reaction.

### 2.10. ZIF Based Materials

**Figure 8:**
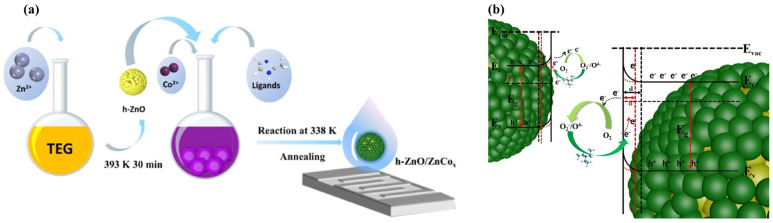
(**a**) Schematic representation of the preparation of h-ZnO/ZnCo_x_ and (**b**) sensing mechanism of TEA using h-ZnO/ZnCo_x_. Reproduced with permission [101].

Yang et al. [99] designed carbon dot (CDs) activated MOF-based gas sensor for TEA detection. The CDs acted as light energy conversion and fluorescence-modulating components whereas ZIF-8(In) provided active sites and structural support. For aqueous TEA detection, CDs@ZIF-8(In) functioned as a dual-emission ratiometric fluorescent sensor showing TEA-dependent emission enhancement at 440 and 610 nm with a detection limit of 1 ppm. For gas detection, the MOF-derived CDs@DZIF-8(In) sensor showed response of 374.6 toward 100 ppm TEA which was 3.5 times higher than pure DZIF-8(In). Li et al. [100] developed hierarchically porous gold decorated ZnO/zeolitic imidazolate framework-8 (Au-ZnO/ZIF-8) hetero-structure for ppb-level triethylamine (TEA) sensing applications. The gas sensing material was prepared by electro-spinning ZnO nanofibers, growing ZIF-8 layer on the ZnO surface, decorating the composite with Au NPs, and then applying interface oxidation whereas retaining the porous ZIF-8 framework. The optimized Au-ZnO/ZIF-8 based gas sensor exhibited response of 1012.5 towards 100 ppm TEA at 235 °C with detection limit of 2.78 ppb and response time of 7 s. The excellent performance was attributed to the porous ZnO/ZIF-8 interface, preserved MOF adsorption sites, and Au-induced catalytic/electronic sensitization. Wei et al. [101] reported Co-doped hierarchical ZnO (Co-doped h-ZnO) core-shell structures derived from ZIF-based self-sacrificing templates (Figure 8a). The sensing materials were prepared by pyrolyzing hierarchical porous ZnO submicrospheres coated with Co-containing ZIF layers. Co doping modulated donor defects, including zinc interstitials and oxygen vacancies, thereby increasing adsorbed oxygen species and active reaction sites. The optimized 15% Co doped h-ZnO sensor delivered high response of 1020 towards 50 ppm TEA. The enhanced sensing behavior was attributed to Co-induced defect engineering, additional electrons, and abundant active oxygen species that accelerated TEA oxidation on the ZnO surface. The mechanism for TEA has been illustrated in Figure 8b.

Fan et al. [102] proposed an electrospinning-assisted strategy to suppress agglomeration of ZIF-67 derived Co_3_O_4_ during calcination and improve TEA sensing. Instead of using directly calcined MOF powders, ZIF-67-derived Co_3_O_4_ polyhedrons were embedded into continuous porous fibers which improved structural stability and retained porosity. Compared with Co_3_O_4_ polyhedrons, the porous fibers showed a 25.6% higher specific surface area, 2.1-fold higher TEA response, and response/recovery times shortened by 53 s. Gao et al. [103] reported amorphous derivative of ZIF-67 as a low-temperature TEA sensing material. By calcining ZIF-67 at 260 °C, the authors obtained amorphous nanocubes with an average size of about 200 nm, a high surface area of 350.2 m^2^/g, and a mesopore ratio of 77.3%. Unlike crystalline Co_3_O_4_ derivatives formed at higher temperatures, the amorphous derivative retained a porous nanocube structure and exhibited more active adsorption sites. The sensor delivered a response of 74.8 towards 100 ppm TEA at 100 °C under 30% relative humidity, along with good selectivity, long-term stability, and response under high humidity. The improved performance was mainly attributed to high adsorbed V_O_, large surface area, and high porosity. Xiao et al. [104] developed an In-doped ZIF-8-based multifunctional TEA sensor capable of both fluorescence detection in water and resistive gas sensing in air. In doped interpenetrating twin ZIF-8 showed TEA-dependent fluorescence enhancement at 450 nm in aqueous solution over 1–100 ppm with detection limit of 1 ppm. After annealing, the ZIF-8-In precursor converted into porous hierarchical ZnO/In_2_O_3_ which functioned as a chemiresistive gas sensor. The ZnO/In_2_O_3_ sensor showed response of 107.7 towards 100 ppm TEA. It is also worthy to mention that oxidation of TEA on MOF-derived sensing materials cannot be described by a single universal reaction pathway. For example, Cu-doped α-Fe_2_O_3_ was proposed to oxidize TEA into N_2_, CO_2_, and H_2_O [66], whereas PdO-Co_3_O_4_-SnO_2_ produced nitrogen-containing products that were more broadly assigned as NO_x_ [94]. In contrast, ZnO/Ag and ZnO/In_2_O_3_ sensors proposed NO_2_ as the final nitrogen-containing product [97,104]. These differences likely arise from variations in surface composition, catalytic activity, oxygen-vacancy concentration, dominant adsorbed oxygen species, and operating temperature. Highly oxidative surfaces may promote the stepwise oxidation of nitrogen-containing intermediates to NO or NO_2_, whereas less oxidative surfaces may favour dealkylation and subsequent formation of N_2_. Despite these differences, the underlying sensing process is similar: TEA reacts with surface-adsorbed oxygen species, releasing electrons back to the semiconductor and changing its resistance. It should also be emphasized that most reported reaction equations are proposed from chemiresistive behaviour rather than direct identification of gaseous products. Therefore, N_2_, NO_x_, and NO_2_ should be considered plausible, material-dependent pathways rather than conclusively established products. Future studies using operando spectroscopy, gas chromatography-mass spectrometry, and isotope-labelling experiments are needed to clarify the actual nitrogen-containing products formed during TEA sensing.

### 2.11. Mo Based Metal Oxides

**Figure 9:**
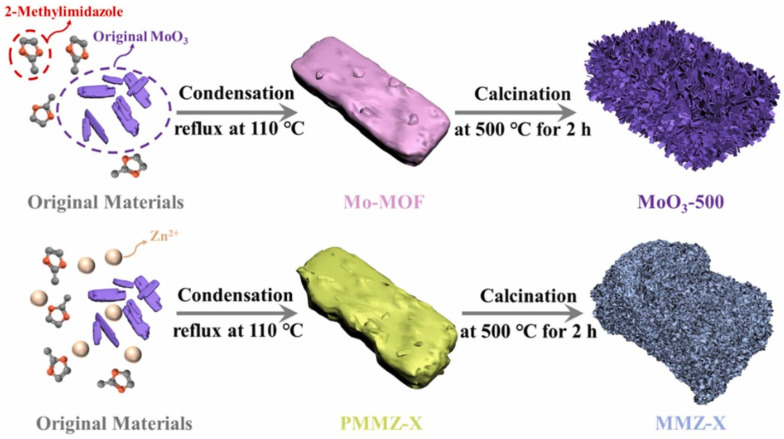
Schematic representation of the construction of MoO_3_-500 and MMZ-X. Reproduced with permission [107].

Zhang et al. [105] investigated the morphology evolution of MOF-derived alpha molybdenum trioxide (α-MoO_3_) for TEA sensing. By pyrolyzing Mo–MOF nanorod precursors at different temperatures, the authors obtained α-MoO_3_ with varied morphologies including nanorods, nanoplates, and microsheets. Among these structures, the MOF-derived α-MoO_3_ nanoplate sensor showed better TEA sensing behavior with a response of 121.1 towards 100 ppm TEA, excellent selectivity, and detection limit of 0.2 ppm. The sensor also displayed a very fast response time of 3 s, although the recovery remained relatively slow at 715 s, reflecting the strong interaction between alkaline TEA molecules and the acidic α-MoO_3_ surface. The enhanced response may be associated with surface morphology and favorable interactions between TEA and lattice oxygen on α-MoO_3_. Liu et al. [106] developed MoO_3_/TiO_2_ (MMT-X) hetero-structures using Mo–MOF and titanium carbide MXene (Ti_3_C_2_T_x_) hybrid precursors for high-performance TEA sensing. During calcination, Mo–MOF was converted into rod-like α-MoO_3_ whereas Ti_3_C_2_T_x_ MXene was transformed into TiO_2_, forming closely connected MoO_3_/TiO_2_ interfaces. The optimized MMT-2 sensor exhibited a high response of 566.7 toward 100 ppm TEA, together with good linearity, fast response/recovery, decent selectivity, repeatability, and stability. The enhanced sensing behavior was associated with porous structure, abundant surface defects, V_O_, and efficient carrier transport at the MoO_3_/TiO_2_ hetero-interface. Liu et al. [107] reported zinc-doped molybdenum metal–organic framework (Mo–MOF)-derived MoO_3_/zinc molybdate (MoO_3_/ZnMoO_4_, MMZ-X) heterostructures for efficient TEA sensing (Figure 9). In this work, Zn^2+^ was introduced in situ into the Mo–MOF precursor through simple reflux-condensation method followed by calcination to form MoO_3_/ZnMoO_4_ hetero-structures.

The optimized MMZ-2 based gas sensor showed high response of 572.3 towards 100 ppm TEA with good selectivity, repeatability, and stability. In another study, Ma et al. [108] designed 1D MoO_3_/ZnMoO_4_/cobalt molybdate (MoO_3_/ZnMoO_4_/CoMoO_4_) hierarchical structures using bi-component MOF-derived strategy. The optimized architecture consisted of MoO_3_ nanobelts as core, uniform ZnMoO_4_ shell and tunable CoMoO_4_ NPs on the outer surface. This multilevel structure offered abundant surface/interface sites, improved electron transport, and enhanced surface adsorption/reaction towards TEA. The optimized sensor showed high response of 505.67 towards 10 ppm TEA at 270 °C with excellent selectivity and long-term stability. The superior sensing performance was attributed to multi-level hetero-junctions, large specific surface area, and efficient surface/interface electron transfer. In another study [109], three-phase bismuth molybdate (BMO) hetero-junction metal oxide semiconductor based composite comprising α-, β-, and γ-BMO phases was also prepared through one-pot MOF-derived synthesis. This sensor demonstrated good selectivity, good long-term stability, and rapid response/recovery times for TEA detection.

### 2.12. Others

**Figure 10:**
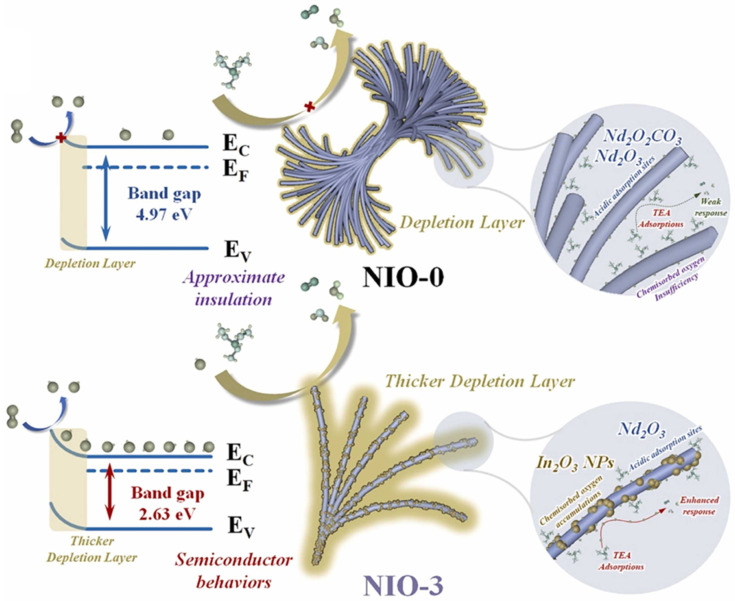
Schematic graph representing the sensing of TEA. Reproduced with permission [113].

Chen et al. [110] constructed bilayer TEA sensors using cobalt MOF-derived Co_3_O_4_ porous sensing films and tin oxide (SnO_2_) catalytic overlayers. The Co-MOF films were grown directly on ceramic substrates and converted into Co_3_O_4_ porous films, avoiding the limitations of conventional powder coating. A thin SnO_2_ over layer was then introduced to improve conductivity and catalytic activity. The optimized SnO_2_/Co_3_O_4_ bilayer sensor worked at room temperature and showed response of 150% towards TEA with fast response/recovery times of 11/16 s and good selectivity. The enhanced performance was ascribed to the controlled bilayer structure, improved electrical conduction and the synergistic role of SnO_2_ as a catalytic over layer and Co_3_O_4_ as the porous sensing layer. Qin et al. [111] reported MOF-derived hollow mesoporous lanthanum ferrite/lanthanum oxide (LaFeO_3_/La_2_O_3_) hetero-structures for high-performance TEA sensing. The MOF-derived route enabled formation of a hollow mesoporous architecture and La_2_O_3_ decorated LaFeO_3_ hetero-junctions. The optimized M-LaFeO_3_-700 sensor delivered a response of about 150 toward 100 ppm TEA at 240 °C which is nearly ten times higher than pristine LaFeO_3_. It also showed rapid response/recovery times of 31/41 s, good reproducibility, long-term stability, high selectivity, and low detection limit. The improved sensing behavior was mainly attributed to faster gas diffusion through the hollow mesoporous structure and improved charge separation at the LaFeO_3_/La_2_O_3_ interface. Wang et al. [112] synthesized ZIF-67 modified SnO_2_ composites (ZSnO_2_) using solvothermal method followed by calcination for TEA detection. Incorporating different amounts of ZIF-67 introduced cobalt derived Co_3_O_4_ into the SnO_2_ matrix forming p-n hetero-junctions and increasing the V_O_ and specific surface area. The optimized ZSnO_2_-60 based gas sensor showed response of 142.6 toward 50 ppm TEA at 170 °C which was about 3.81 times higher than pure SnO_2_. The ZSnO_2_ sensors also showed lower working temperature, good selectivity, and repeatability. The enhanced performance may be associated to V_O_ enrichment, enlarged active surface, and Co_3_O_4_/SnO_2_ hetero-interfaces that promoted charge transfer and surface reaction with TEA. Jin et al. [113] introduced an ion-insertion strategy to transform neodymium oxycarbonate (Nd_2_O_2_CO_3_) based materials from approximate insulators into semiconducting TEA-sensing nanocomposites. In^3+^ insertion into MOF-76(Nd) derived Nd_2_O_2_CO_3_ reduced the band gap, improved electron transfer, improved V_O_ and Lewis acidic sites for TEA adsorption. The optimized sensor showed a response of 167.94 toward TEA at 160 °C with fast response/recovery times of 5/42 s. The illustration for the TEA sensing is shown in Figure 10.

The proposed sensor also displayed interesting selectivity for the monitoring of TEA. The improved TEA selectivity may arises from the combined effects of preferential adsorption and surface-reaction kinetics rather than from a single material property. The electron-rich nitrogen atom makes TEA a strong Lewis base, favoring its interaction with Lewis-acidic metal centres and oxygen-deficient sites. This behavior is supported by the strong affinity of alkaline TEA for acidic α-MoO_3_ surfaces [105] and the enhanced TEA adsorption at Lewis-acidic and oxygen-vacancy-rich sites in In^3+^ inserted Nd_2_O_2_CO_3_ [113]. DFT calculations further indicate that engineered Ti_3_C_2_T_x_/Co-BDC interfaces strengthen TEA adsorption and interfacial charge transfer [62]. Following adsorption, Ag assisted oxygen activation and catalytic spillover accelerate TEA oxidation and amplify the resistance change relative to less reactive interfering gases [97]. Therefore, selective TEA detection requires an appropriate balance of surface acidity, accessible adsorption sites, defect density, and catalytic activity. The gas sensing performance of the various MOF-derived materials towards the quantification of TEA have been summarized in Table 1.

Table 1 reveals substantial progress in the development of MOF-derived materials based TEA sensors. It can be observe that SnO_2_/TiO_2_ shows the highest response (3525.2 at 10 ppm). The Au-ZnO/ZIF-8, α-Fe_2_O_3_, and Au/In_2_O_3_ also exhibit strong responses. However, several of these gas sensing systems require high temperatures or show slow recovery. In contrast, Ru/Mo co-doped Co_3_O_4_ and Ga-doped Co_3_O_4_ exhibit more balanced combination of response, rapid response/recovery, and moderate operating temperature. The Ti_3_C_2_T_x_/Co-BDC composite and SnO_2_/Co_3_O_4_ bilayer are particularly notable for relatively low-temperature operation, fast kinetics, and sub-ppm detection limits. Overall, heterojunction construction, metal doping, noble-metal sensitization, and hierarchical porosity clearly improve TEA adsorption and interfacial charge transfer, but frequently introduce trade-offs between sensitivity, recovery rate, operating temperature, and stability. Future studies should therefore prioritize standardized response metrics, humidity tolerance, long-term stability, and low-temperature selectivity rather than focusing solely on maximum response values.

## 3. Conclusions, Limitations, and Perspectives

MOF-derived materials provide a versatile gas sensing platform for the detection of TEA by integrating hierarchical porosity, tunable composition, defect chemistry, and interfacial charge modulation. As per the summarized literature, most effective improvements arise from coupling accessible gas-diffusion pathways with controlled oxygen vacancies, heterojunctions, catalytic dopants, and conductive components. However, the MOF precursor does not inherently ensure TEA selectivity. Selectivity is also influenced by the surface chemistry of the derived phase, adsorption energetics, catalytic activity, operating temperature, and reaction kinetics. Thus, the rational control of structure–defect–interface relationships, rather than maximizing surface area alone, should guide future material design.

### 3.1. Current Limitations

Limited intrinsic selectivity. MOF-derived pristine material-based chemiresistive gas sensors may suffer from low response towards TEA.

Dependence on controlled testing conditions. Most studies evaluate sensing performance using single gases under laboratory conditions. The effects of humidity, temperature variation, complex gas mixtures, sensor poisoning, and long-term stability remain insufficiently examined.

High power consumption. Many MOF-derived material-based TEA sensors still require external heating and relatively high operating temperatures, which may restrict their use in portable and wearable devices.

Insufficient reproducibility and standardization. Differences in response definitions, gas concentrations, humidity levels, device configurations, and testing protocols make direct comparison among reported sensors difficult. Batch-to-batch reproducibility and sensor-to-sensor variations are also rarely reported.

Lack of depth regarding mechanistic understanding. The roles of oxygen vacancies, catalytic sites, adsorption strength, reaction intermediates, and N-containing species are often not described in depth. More direct experimental evidence is required to establish reliable structure–mechanism–performance relationships.

### 3.2. Emerging Directions

AI-assisted gas recognition. Artificial intelligence (AI), machine learning (ML), and deep learning (DL) may interpret complex sensor signals and improve gas classification, concentration estimation, drift correction, and interference compensation. 

Self-heated and low-power devices. Self-heating operation, localized Joule heating, and microelectromechanical system platforms remain underexplored for MOF-derived gas sensors. These strategies may reduce power consumption, shorten response times, and facilitate portable sensing.

Wearable and flexible sensors. The integration of MOF-derived sensing materials with flexible electrodes, textiles, polymers, and wireless systems is promising for personal exposure monitoring and occupational safety. Future studies should address mechanical durability, perspiration, humidity interference, and repeated bending.

Mechanism-guided material design. Operando spectroscopy and theoretical calculations may be combined to identify active sites and clarify TEA oxidation pathways. Such understanding will support the rational control of porosity, defects, catalytic sites, and hetero-interfaces.

Real time monitoring and standardization. Future sensors should be tested in humid air, mixed-gas atmospheres, food-spoilage environments, and industrial settings. Standardized performance metrics, scalable fabrication, long-term stability, and energy-consumption analysis will be essential for practical deployment.

## Figures and Tables

**Table 1:** Gas sensing performance of the reported MOF-derived materials for TEA detection.

Materials	Response	Concentration (ppm)	Response Time (s)	Recovery Time (s)	Limit of Detection (LOD) (ppm)	Stability	Temperature (°C)	Ref.
Cr_2_O_3_/TiO_2_-X	450.01	100	26	112	-	30 days		[61]
Ti_3_C_2_T_x_/Co-BDC	71	50	11	20	0.085	7 week	100	[62]
SnO_2_/TiO_2_	3525.2	10	1175	-	10 ppb	14 days	61	[63]
5 at % Fe-doped Co_3_O_4_	21	100	32	34	5	-	220	[64]
Co-MOF	1.14	400	85	70	4	40 days	25	[65]
α-Fe_2_O_3_	527	50	20	8	45.05 ppb	20 days	180	[67]
Se/α-Fe_2_O_3_	20.8	100	4	94	0.2	-	275	[68]
Bow-like Ga-doped Co_3_O_4_ structure	108	50	3	15	0.1	35 days	180	[69]
Flower-like Mo-doped Co_3_O_4_	92	50	92	33	-	30 days	180	[70]
Al/Mo-doped Co_3_O_4_ tetrahedrons	132	100	4	36	0.5	35 days	160	[72]
Ru/Mo co-doped Co_3_O_4_ hollow microtubes	126	100	5	7	-	30 days	160	[73]
Co_3_O_4_/Fe_2_O_3_	21.2	100	13	15	1	-	199	[74]
In_2_O_3_/ZnO	44.6	100	14	36	-	-	100	[75]
Mesoporous flower-like ZnO/NiO heterostructure	49.8	200	11	130	-	70 days	200	[76]
Hierarchical kiwifruit-like ZnO/ZnFe_2_O_4_	40.15	100	32	41	-	30 days	200	[77]
GaFeO_3_	7.4	200	9	49	-	31 days	200	[78]
In_2_O_3_-NiO hollow sphere	33.9	100	-	-	0.5	26 days	200	[79]
Nd_2_O_3_-ZnO nanocages	360.09	100	1	628	150 ppb	45 days	157	[80]
MOF-ZnO/ZnFe_2_O_4_	7.6	100	1	9	-	30 days	170	[82]
NV@NiO-2	43.7	100	88	127	4.5 ppb	13 days	240	[83]
NiO/ZrO_2_-2	32.3	100	55	83	7.2 ppb	13 days	240	[84]
Bimetallic MOF-derived Ni/SnO_2_ composites	124	50	-	-	128 ppb	30 days	170	[85]
Cu/C-ZnO	225	100	17	38	-	25 days	280	[86]
g-C_3_N_4_/Co_3_O_4_	48.52	100	47	339	1	45 days	119	[87]
In_2_O_3_ nanocubes	21.6	100	1	28	100 ppb	30 days	350	[88]
Porous lacunaris In_2_O_3_	226	5	9	36	-	40 days	120	[89]
Ag-ZnO	430	100	9	49	1	30 days	225	[92]
PdO-Co_3_O_4_-SnO_2_-3	11	20	21	64		21 days	240	[94]
PdO-ZnO-In_2_O_3_ nanofibers	386	50	1	740	0.1	30 days	250	[95]
Pd/PdO@ZnO-ZnO	258	50	1	234	3	30 days	275	[96]
ZnO/Ag	293.8	10	64	28	46.5 ppb	15 days	200	[97]
Au/In_2_O_3_	476.3	100	32	279	12 ppb	30 days	200	[98]
CDs@D_ZIF-8(In)_	374.6	100	16	90	1	15 days	160	[99]
Au-ZnO/ZIF-8	1012.5	100	7	163	2.78 ppb	-	235	[100]
Porous Co_3_O_4_ fibers	34.5	100	-	-	100 ppb	30 days	160	[102]
Amorphous ZIF-67 derivative	74.8	100	125	88	-	30 days	100	[103]
*α*-MoO_3_ nanoplates	121.1	100	3	715	0.2	15 days	250	[105]
SnO_2_/Co_3_O_4_ bilayer films	150% ^a^	100	11	16	84 ppb	-	25	[110]
MOF-drived hollow mesoporous LaFeO_3_/La_2_O_3_	150	100	31	41	0.5	30 days	240	[111]
ZSnO_2_-60 composites	142.6	50	-	-	-	30 days	170	[112]

^a^ Response (%) = (Rg − Ra)/Ra × 100.

## Data Availability

No new data was generated in this study.
